# Implications of human–wildlife conflict on the diet of an endangered avian top predator in the northern Andes

**DOI:** 10.1038/s41598-024-63947-3

**Published:** 2024-06-06

**Authors:** Juan Sebastián Restrepo-Cardona, Sebastián Kohn, Luis Miguel Renjifo, Juan D. Vásquez-Restrepo, Santiago Zuluaga, Félix Hernán Vargas, Fabricio Narváez, Luis A. Salagaje, Abel Recalde, Erik Camilo Gaitán-López, Andy Salazar, Vanessa Hull

**Affiliations:** 1 Department of Wildlife Ecology and Conservation, University of Florida, Gainesville, USA; 2 Fundación Cóndor Andino, Quito, Ecuador; 3 Departamento de Ecología y Territorio, Facultad de Estudios Ambientales y Rurales, Pontificia Universidad Javeriana, Bogotá, Colombia; 4 Laboratorio de Herpetología, Museo de Zoología “Alfonso L. Herrera”, Universidad Nacional Autónoma de México, Ciudad de México, Mexico; 5 Colaboratorio de Biodiversidad, Ecología y Conservación (INCITAP-CONICET/FCEyN-UNLPam), Santa Rosa, Argentina; 6 Fundación Proyecto Águila Crestada-Colombia, Manizales, Colombia; 7 The Peregrine Fund, Galápagos, Ecuador; 8 Universidad Surcolombiana, Neiva, Colombia

**Keywords:** Conservation biology, Ecology

## Abstract

Conflicts between rural people and the Endangered Black-and-chestnut Eagle (*Spizaetus isidori*) are a prominent conservation concern in the northern Andes, as at least 60 eagles were poached between 2000 and 2022 in response to poultry predation. Here, we conducted direct observations to analyze the Black-and-chestnut Eagle diet and evaluated how forest cover affects the feeding habits of the species during nestling-rearing periods in 16 nests located in different human-transformed Andean landscapes of Ecuador and Colombia. We analyzed 853 prey items (46 species) delivered to nestlings. We used Generalized Linear Models to test whether the percent forest cover calculated within varying buffer distances around each nest and linear distances from the nest to the nearest settlement and pasture areas were predictors of diet diversity and biomass contribution of prey. Forest cover was not a factor that affected the consumption of poultry; however, the eagle regularly preyed on chickens (*Gallus gallus*) (i.e., domestic Galliformes) which were consumed by 15 of the 16 eagle pairs, with biomass contributions (14.57% ± 10.55) representing 0.6–37% of the total prey consumed. The Black-and-chestnut Eagle is an adaptable generalist able to switch from mammalian carnivores to guans (i.e., wild Galliformes) in human-dominated landscapes, and eagles nesting in sites with low forest cover had a less diverse diet than those in areas with more intact forests. Management actions for the conservation of this avian top predator require studies on the eagle’s diet in areas where human persecution is suspected or documented, but also maintaining forest cover for the wild prey of the species, development of socio-economic and psychological assessments on the drivers behind human-eagle conflicts, and the strengthening of technical capacities of rural communities, such as appropriate poultry management.

## Introduction

Anthropogenic habitat conversion forces predators to adapt to feeding on alternative prey species to meet their basic metabolic needs^1–4^. In landscapes transformed by people, predators usually modify their diet by feeding on domesticated animals, which are more available and may require a lower energetic expenditure in anthropized environments^5–7^, leading to human–wildlife conflicts^8–11^. Predators' survival in human-modified habitats will depend not only on the flexibility of their diet and the strategies used to obtain food^12,13^ but also on anthropogenic pressures (e.g., habitat loss and degradation, poaching, depletion of prey)^14,15^, people's attitudes towards the species^16^, and human–wildlife interactions that can be altered by changing landscape factors^17^. Conflicts between people and predators due to predation on domestic animals can lead to the human persecution of species that could be rapidly eliminated at the landscape scale^5,18^.

**Figure 1:**
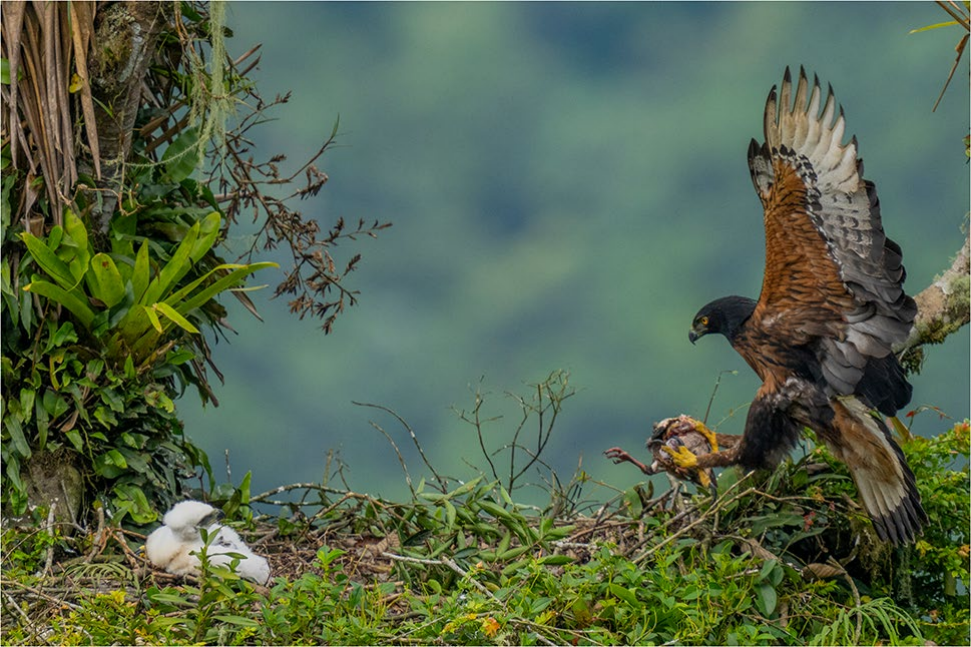
Black-and-chestnut Eagle (*Spizaetus isidori*) delivering an Andean Guan (*Penelope montagnii*) to the nestling in a nest in Ecuador (photo: Jaime Culebras).

The Black-and-chestnut Eagle (*Spizaetus isidori*; Fig. 1) is one of the largest avian top predators of the Andes, weighing between 1.5 and 4 kg^19,20^. It is distributed across Andean montane forests from the north of Colombia and northwestern Venezuela to northern Argentina, in an elevation range between 500 and 3500 m above sea level^21^. Due to anthropogenic threats, mainly through habitat loss, shooting, and capture, but also by electrocution on power lines, illegal trafficking, and collision with vehicles^8,9,22,23^, the number of individuals in the wild probably does not exceed 1000 adults and is declining. This species is therefore listed as Endangered globally according to the International Union for Conservation of Nature^24^. The conservation status of the Black-and-chestnut Eagle is of particular concern in the northern Andes (Ecuador and Colombia), one of the most important population strongholds of the species^22,25^, where human-eagle conflicts are a prominent conservation issue^8,10,11^. Between 2000 and 2022, at least 60 Black-and chestnut Eagles were poached as retaliation or as a preventive measure against poultry predation^9^.

The feeding habits of Black-and-chestnut Eagles have been poorly documented. Only three studies have quantitatively described the diet of the species through the analysis of prey items delivered to nestlings, which account for four Black-and-chestnut Eagle pairs in Colombia and one pair in Argentina^7,26,27^. The Black-and-chestnut Eagle feeds on a wide variety of prey, including large-size birds, arboreal mammals, and reptiles. In Colombia, poultry was a relatively frequent prey in the diet of the species, representing 9–36% of the total prey consumed^7,26^, while in Ecuador, there is no empirical data on the eagle's diet, and it is not documented whether the species is responsible for poultry predation. Diet descriptions provide useful information on the local prey-based composition of predators, but such studies may offer little to improve the effective management of species^28^. Range-wide studies are important for understanding general patterns and informing policy and management strategies for the conservation of predators^18^.

To study the Black-and-chestnut Eagle diet and analyze how forest cover affects the species' feeding habits during nestling-rearing periods in the northern Andes, our aims were twofold. We first evaluated the main prey items of the Black-and-chestnut Eagle, by analyzing the relative frequency and biomass contribution of prey delivery to nestlings in 16 nests located in different human-transformed Andean landscapes of Ecuador and Colombia. Secondly, we examined the influence of forest cover on changes in the diet of Black-and-chestnut Eagles using the niche breadth index and quantitative analyses of species richness and biomass contribution of prey. We predicted that even though wild birds and mammals are the predominant prey in the diet of the eagle, poultry is an important source of food for the species^7,26,27^. We also predicted that the species is an adaptable generalist able to switch prey in different landscapes and diet diversity would differ between pairs nesting in sites with low forest cover and those nesting in more intact forests^2,4^. The results of this study may help decision-makers focus on management and conservation strategies based on scientific evidence to mitigate human-Black-and-chestnut Eagle conflicts.

## Methods

### Study area

**Figure 2:**
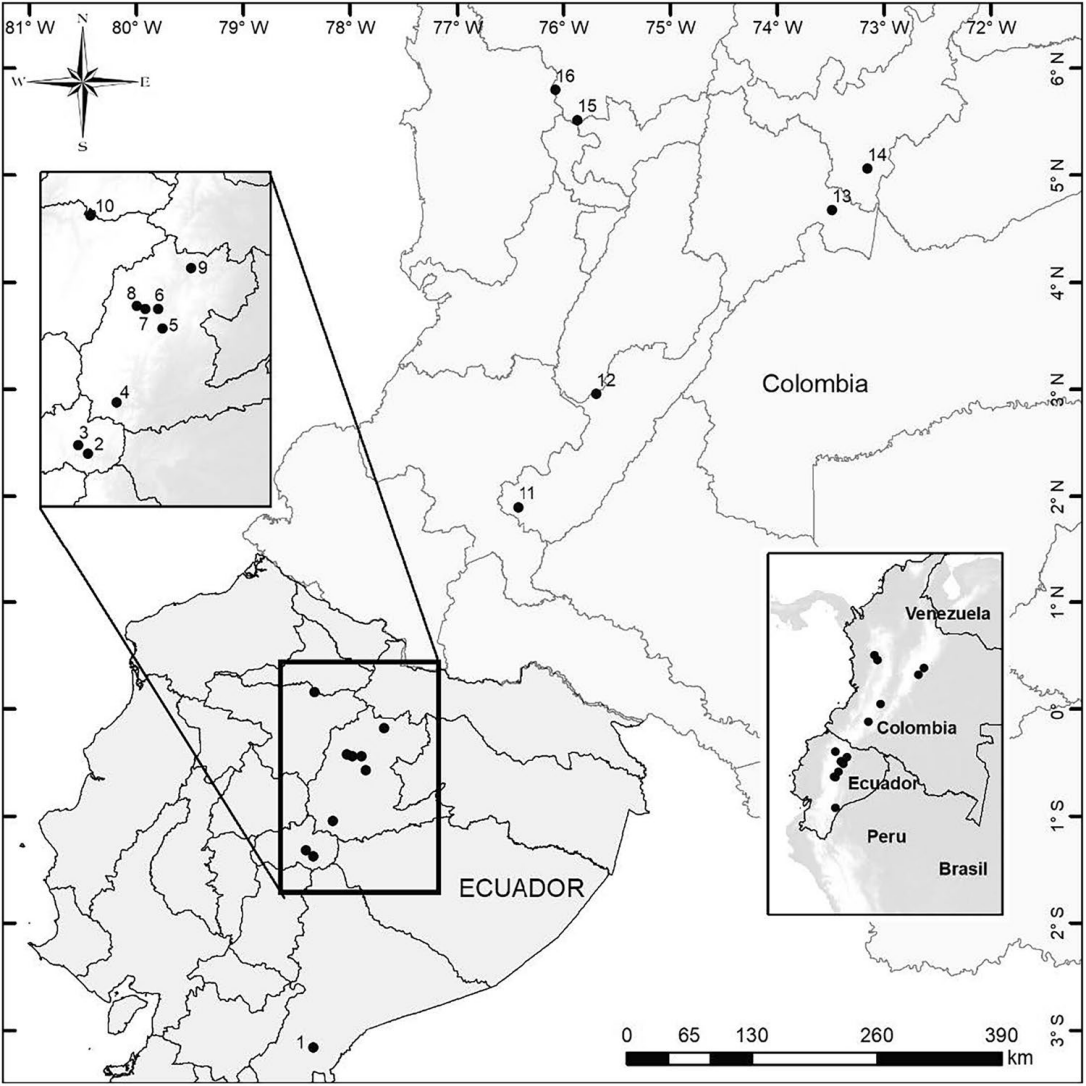
Map of the locations of 16 Black-and-chestnut Eagle (*Spizaetus isidori*) nests in the northern Andes (Ecuador and Colombia). (1) Chimandáz, (2) Río Blanco, (3) El Triunfo, (4) Zuñag, (5) Arenillas, (6) Parada Larca, (7) Quijos Huaico, (8) Cuyúja, (9) El Salado, (10) Atahualpa, (11) San Agustín, (12) Santa María, (13) Gachalá, (14) Campohermoso, (15) Jardín, and (16) Ciudad Bolívar. The map was created using ArcGIS 10.8 software (URL: https://www.arcgis.com/index.html).

This study was conducted in 16 Black-and-chestnut Eagle nests and their surrounding landscapes in the northern Andes (Fig. 2). The 16 nests included 10 in Ecuador and six in Colombia at an altitudinal range from 1578 to 2727 m above sea level (Supplementary Table 1). The eagle nests and their surrounding landscapes consist of varying proportions (12.8–98.2%) of forest cover, heterogeneous agricultural areas, cattle pastures, herbaceous or shrubby vegetation, transitional or permanent crops, man-made bodies of water, urban areas, and industrial zones (Supplementary Table 2).

The Andean tropical and subtropical forest is one of the most severely threatened biodiversity hotspots worldwide^29^. The Andean mountain forests in Ecuador and Colombia have been extensively degraded due to human disturbances such as cattle ranching, agriculture, forest clearance, illicit crops, and human population growth^30,31^. For instance, in Colombia, the Black-and-chestnut Eagle has historically lost 61% of its natural habitat, and in 10 years, it lost 6.8% of its habitat^22^.

### Data collection and acquisition

To evaluate the feeding habits of the Black-and-chestnut Eagle, we carried out systematic observations during nestling-rearing periods at 12 nests between 2017 and 2023. Breeding raptors are relatively easier to observe for documenting prey captured because of their low mobility^32^. Observations were conducted in October 2017 in Arenillas, between May and September 2018 in Zuñag, between October 2018 and January 2019, and between July and October 2021 in Atahualpa, between April and June 2019 in San Agustín, between August and October 2019 and between July and November 2021 in Río Blanco, between May and July 2021 in Santa María, between June and September 2021 and between June and July 2023 in Quijos Huaico, between October and December 2022 in Parada Larca, between April and July 2022 in Cuyúja, between June and August 2022 in Chimandáz, between July and October 2022 in El Triunfo, and between February and May 2022 and between May and September in El Salado.

Direct observations at all nests were performed by trained technicians, using binoculars (10 × 42 and 10 × 50), telescopes (20–60 × 60 and 20–60 × 65), and photographic cameras, from high observation points at a horizontal distance of approximately 50 m from each nest. Observations were made between 0600 and 1800 h at each of the nests. We completed our dataset with four additional nests with data available in the literature, in which prey items were also recorded during nestling-rearing periods^7,26^. This bibliographic research was performed in Google Scholar and Scopus using the keywords: Spizaetus isidori, Black-and-chestnut Eagle, águila andina, águila crestada, and águila real de montaña, combined with diet, feeding habits, dieta, hábitos de alimentación, Ecuador, and Colombia.

Prey items were identified to the finest possible taxonomic level from photographs using bird, mammal, and snake guides^33–37^. Diet composition was expressed as the frequency of each type of prey relative to all types of prey. We defined prey biomass delivery rates by estimating the prey biomass delivered to each nest. Mean body masses of prey species were obtained from the literature^19,33,35,38^. Unidentified prey items were not considered for prey biomass calculation.

To define the landscape composition at each locality, the nests were assumed as the central point, and to explore the effects of the spatial scale, buffers from 0.5 to 4 km, increasing by 0.5 km were generated around them. The different types of land cover were identified (Supplementary Table 2) using Geographic Information System (GIS) tools, based on 30 m resolution Landsat images and using the CORINE Land Cover definitions adapted for Colombia^39^ and extrapolated for Ecuador. The shortest distances between the 16 nests were recorded between the nests in Quijos Huaico and Cuyuja (6.2 km), between the nests in El Triunfo and Río Blanco (9.2 km), and between the nests in Quijos Huaico and Parada Larca (9.4 km), where systematic monitoring of the nests during 2021 and 2023 allows us to guarantee they were different eagle pairs rearing their nestlings. We considered each nest as an independent unit. We also obtained the distance to each nest's nearest settlement and pasture areas because pairs of eagles nesting within forest territories could access poultry and open-habitat prey in nearby settlement and pasture areas, respectively^1,7^. We used images obtained during the nestling-rearing periods in which direct observations were conducted in each nest (Supplementary Table 2).

### Data analysis

We estimated the number of prey samples needed to adequately represent the feeding habits of the Black-and-chestnut Eagle by rarifying a subsampled dataset of prey items 1000 times^28^. We included only the seven species representing 5% or more of the relative biomass contribution. Accounting for all seven species would require more than 80 samples, which exceeds the available data for most of the sampled nests. However, it is possible to account for six of those seven prey items (> 85%) with only 20 samples, so we selected 20 as the most optimal value.

To evaluate the diet diversity of species, the Levins’ standardized food-niche Breadth^40^ index was calculated: B_sta_ = B − 1/(n − 1), where B is the Levins’ index (B = 1/Σp_i_^2^), pi is the percentage of each prey category, and n is the total number of prey categories^41^. The values of this index range from 0 (minimum niche breadth, which implies maximum selectivity) to 1 (maximum niche breadth, minimum selectivity)^42^. The biomass contribution of prey species per nest was calculated using Marti’s index^43^: B_i_ = 100 [(Sp_i_ Ni)/Σ (Sp_i_ N_i_)], where Sp_i_ is the weight of species i, N_i_ is the number of individuals of species i consumed, and B_i_ is the total biomass percentage contributed by species i.

We constructed Generalized Linear Models (GLM) with a Gaussian error structure to examine the effects of forest cover on the Black-and-chestnut Eagle feeding habits. Our response variables were the standardized Levin’s index values, prey species richness, and biomass contribution of prey. Our explanatory variables included the percentage of non-forest and linear distances from the nest to the nearest settlement and pasture areas during the nestling-rearing periods (Supplementary Table 2). We added distance to the nearest settlement and pasture areas as covariates. We tested for scale dependence using non-forest values calculated within varying buffer distances at 0.5 km intervals ranging from 0.5 to 4 km from the nest. However, we present only the results of the 2 km buffer distance because empirical data reveal that the mean scale of the effect of neotropical diurnal raptors is 1633 ha (landscapes of 2279-m radius)^44^, and our results for the standardized Levin’s index showed significance at this scale. In addition, to examine the association between the prey items and nests located in Ecuador and Colombia, we performed a simple correspondence analysis. All tests were performed using R software version 2.1^45^. We considered the results to be statistically significant when p < 0.05.

## Results

We analyzed 853 prey items recorded in the 16 Black-and-chestnut Eagle nests. Fourteen prey items were recorded in Arenillas, 42 in Zuñag, 35 in Atahualpa, 37 in San Agustín, 40 in Río Blanco, 24 in Santa María, 78 in Quijos Huaico, 31 in Parada Larca, 80 in Cuyúja, 22 in Chimandáz, 72 in El Triunfo, and 117 in El Salado. Whereas 25 prey items were recorded in Campohermoso, 105 in Gachalá, 75 in Jardín, and 56 in Ciudad Bolívar^7,26^. The mean number of prey per nest was 53.3 ± 31.1 (Supplementary Table 3).

In total, 674 prey items (79%) were identified to species level and 796 (93.3%) to class. Of the prey items identified to species, 85.9% were wildlife and 14.1% were poultry, domestic Galliformes mainly chickens (*Gallus gallus*) and one Turkey (*Meleagris gallopavo*). The Black-and-chestnut Eagle consumed 46 vertebrate species with weights ranging from 0.07 kg of the Lyre-tailed Nightjar (*Uropsalis lyra*) to 6.8 kg of the Common Woolly Monkey (*Lagothrix lagothricha*). The mean body mass of prey species was 1.46 ± 1.88. The standardized Levins’ index values of the Black-and-chestnut Eagle ranged from 0.19 to 0.88. The mean Levins’ index value was 0.49 ± 0.20 (Supplementary Table 3).

Numerically, four top-ranking species represented 54.22% of all prey delivered to nestlings: Sickle-winged Guans (*Chamaepetes goudotii*) (13.30 ± 13.49 ind.) representing 15.59%, Red-tailed Squirrels (*Sciurus granatensis*) (11.36 ± 6.86 ind.) representing 14.60%, Andean Guans (*Penelope montagnii*) (18.50 ± 13.22 ind.) representing 13.01%, and chickens (6.27 ± 6.30 ind.) representing 11.02%. In terms of biomass contribution per nest, the diet of the Black-and-chestnut Eagle was mainly comprised of South American Coatis (*Nasua nasua*) (38.88% ± 19.95) representing 17.5–66.3%, Sickle-winged Guans (19.97% ± 12.18) representing 3.1–44.1%, chickens (14.57% ± 10.55) representing 0.6–37%, and Andean Guans (31.33% ± 24.05) representing 2.7–68.9% of the total prey consumed (Supplementary Table 3).

**Figure 3:**
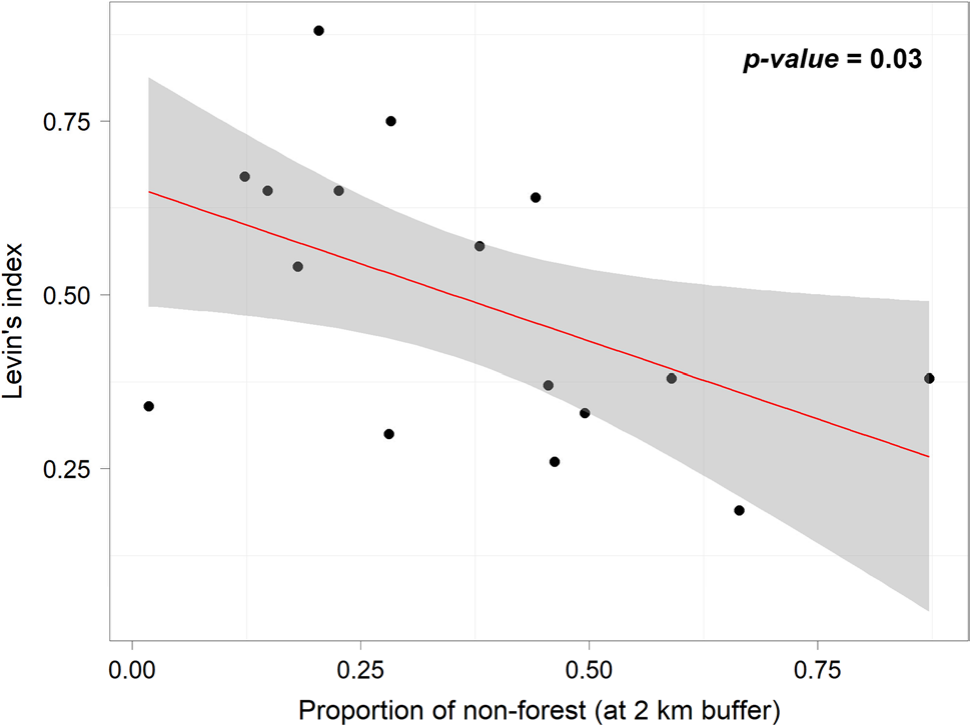
Effects of forest cover (2 km buffer) on the diet composition of Black-and-chestnut Eagles (*Spizaetus isidori*) in 16 nests in the northern Andes (Ecuador and Colombia).

Neither the percentage of non-forest nor the distances to settlement and pasture areas were significant predictors of the richness of consumed species (p-value > 0.05) (Supplementary Table 4). Levins’ standardized food-niche breath index was not significantly predicted by distances to settlement and pasture areas but by the percentage of non-forest at the scale of 2 km (p-value < 0.05). Percent non-forest accounted for between 20 and 31% of the observed variation in the Levins’ standardized food-niche breadth index between nests (Supplementary Table 5). Standardized Levin index values also appeared to decrease as the proportion of forest cover decreased. The dietary breadth of the eagle was lower in areas of lower forest cover (Fig. 3).

**Figure 4:**
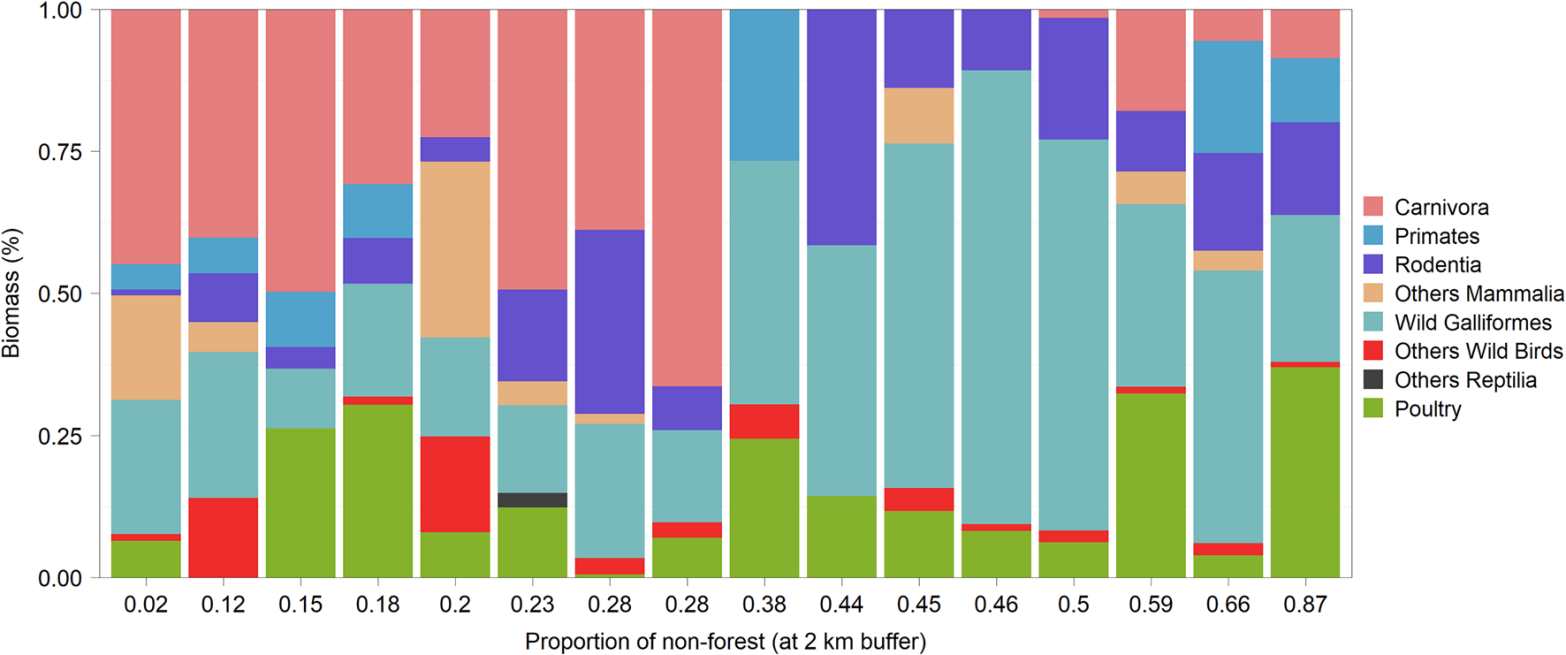
Biomass contribution of different prey items delivered in 16 Black-and-chestnut Eagle (*Spizaetus isidori*) nests in the northern Andes (Ecuador and Colombia). Each column represents a nest, ordered from the lowest to the highest percentage of non-forest within a 2 km buffer around each nest site. Poultry (i.e., domestic Galliformes) includes chickens (*Gallus gallus*) and one Turkey (*Meleagris gallopavo*).

**Figure 5:**
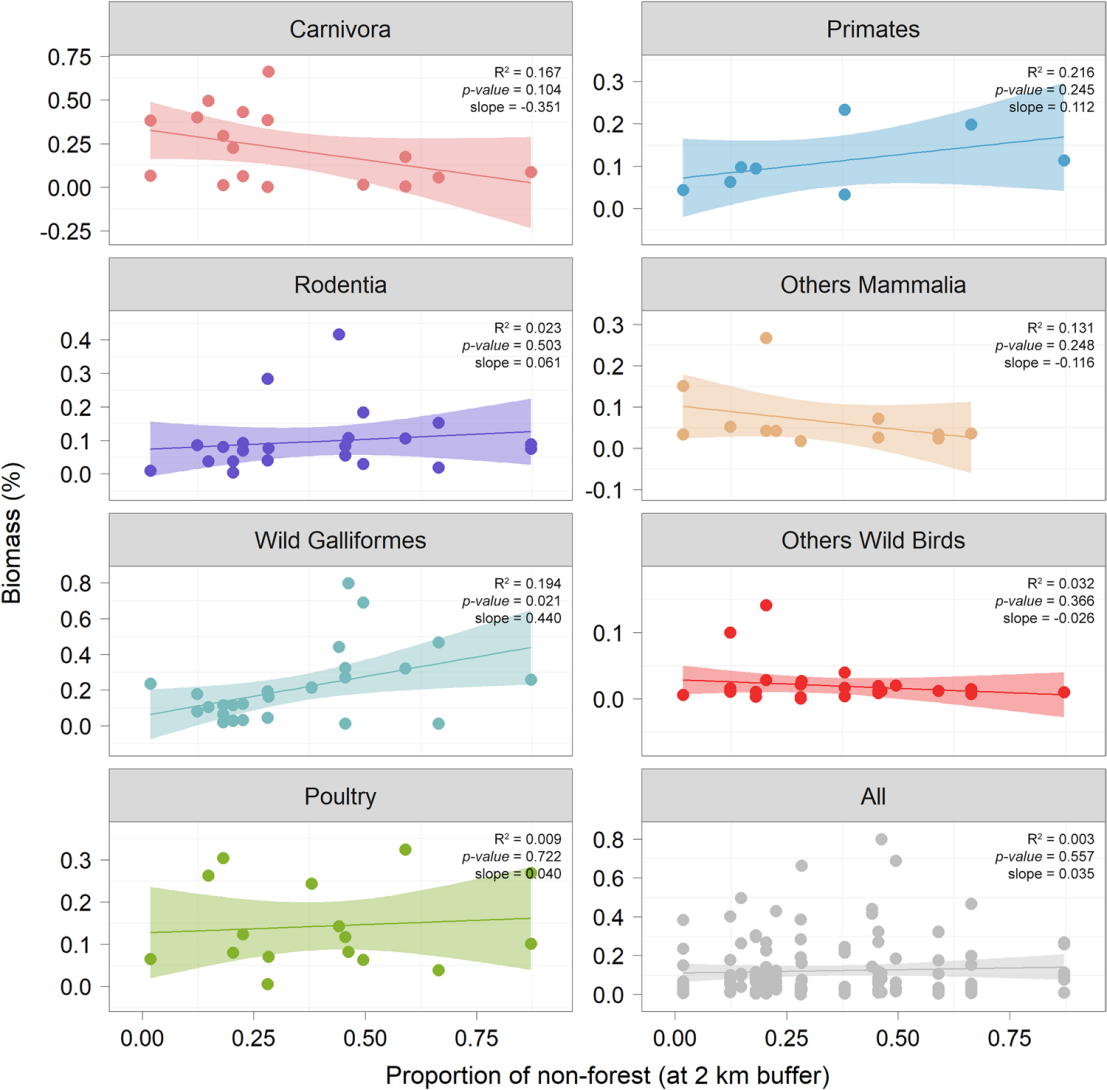
Coefficient values and performance metrics for the relationship between the proportion of non-forest at 2 km buffer and the proportion of consumed prey biomass by order in 16 Black-and-chestnut Eagle (*Spizaetus isidori*) nests in the northern Andes (Ecuador and Colombia). Poultry (i.e., domestic Galliformes) includes chickens (*Gallus gallus*) and one Turkey (*Meleagris gallopavo*).

Forest cover was not a factor that significantly affected the consumption of poultry (i.e., domestic Galliformes). However, we observed a pattern of reduction in the amount of biomass contributed by carnivorous mammals (Order Carnivora) above 30% reduction in forest cover, primarily being replaced by guans (i.e., wild Galliformes) (Fig. 4). We also detected a higher proportion of primates in the diet of the eagle in sites with low forest cover (Fig. 5).

**Figure 6:**
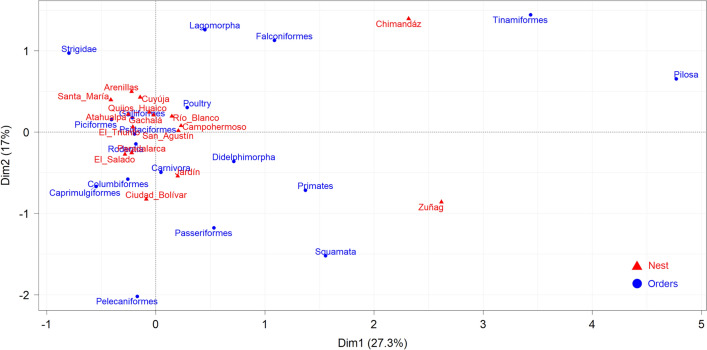
Correspondence analysis for the 16 Black-and-chestnut Eagle (*Spizaetus isidori*) nests analyzed in the northern Andes (Ecuador and Colombia) and the prey taxonomic orders.

Furthermore, the correspondence analysis suggests no particular association between the prey items and nests located in Ecuador and Colombia, except for Chimandáz and Zuñag localities due to the presence of Tinamiformes and Pilosa orders. Moreover, the presence of Primates, Logomorpha, Falconiformes, Pelecaniformes, and Squamata, far from being common prey items recorded in the diet of the Black-and-chestnut Eagle in most nests, seems to be particular prey items in some of them (Fig. 6).

## Discussion

We found that the Black-and-chestnut Eagle regularly eats poultry, mainly chickens, in the northern Andes (Fig. 4, Supplementary Table 3). Eagles nesting in sites with low forest cover had a less diverse diet and showed lower values of Levin’s food-niche breadth index (Fig. 3). The species is an adaptable generalist able to switch from mammalian to wild bird prey in human-transformed landscapes. Habitat loss and potential wild prey depletion are probably pushing the eagle to increasingly rely on alternative prey compared to more intact forests. Our findings show that as forest cover decreased in the breeding territories of the species, the importance of mammalian carnivores (e.g., coatis) in its diet also decreased, but the importance of guans increased (Fig. 5).

Poultry was the fourth most frequent prey in the diet of the Black-and-chestnut Eagle (11.02%; 6.27 ± 6.30 ind.) (Supplementary Table 3). This predation rate on domestic animals is relatively high compared to those recorded for other raptor species worldwide. For instance, in the Neotropics, for the Ornate Hawk Eagle (*S. ornatus*) chickens made up 3.3% of its diet^46^, and for the Chaco Eagle (*Buteogallus coronatus*) goats (*Capra hircus*) represented only 0.2% of its diet^47^. In Africa, domestic animals such as chickens, goats, rabbits (*Oryctolagus cuniculus*), and domestic cats (*Felis domesticus*), comprised 6% of the identifiable prey of the Crowned Eagle (*Stephanoaetus coronatus*)^48^. In Asia, the Philippine Eagle (*Pithecophaga jefferyi*) also consumed chickens (10%), domestic cats (3%) and dogs (*Canis domesticus*) (4%)^49^. In Europe, poultry was a relatively frequent prey in the diet of the Bonelli's Eagle (*Hieraaetus fasciatus*) (frequency: 42.7%), representing up to 37.7% of the total biomass of prey consumed^6^. In the Black-and-chestnut Eagle breeding territories studied in the northern Andes, most poultry owners do not keep their chickens inside coops or protected from avian top predators, making them more vulnerable^8^, but it is unknown whether chicken owners consider that predation rates can be assumed or the cost to design protection is higher than the benefits.

Habitat conversion increases the frequency of interaction between people and forest species^5^, such as the Black-and-chestnut Eagle. Ninety-seven percent of the global distribution range of this species has been impacted by anthropogenic threats^14^, particularly in Colombia, where the eagle has lost 61% of its natural habitat^22^. Forest cover was not a factor that significantly affected the consumption of poultry; however, chickens were consumed by 15 of the 16 Black-and-chestnut Eagle pairs analyzed, with biomass contributions (14.57% ± 10.55) representing 0.6–37% of the total prey consumed (Fig. 4, Supplementary Table 3). The relatively high consumption of poultry indicates that with relative frequency, the eagle forages in rural Andean landscapes of Ecuador and Colombia, where they are at high risk of being hunted^8–11^. Predation of poultry may result in competition between local farmers and Black-and-chestnut Eagles since chickens are the most abundant domestic bird in the northern Andes^50^ and represent an important food source for the species in this geographical region.

Our study adds evidence suggesting that changes in forest cover can lead to variations in the availability of prey eaten by predators^2,51^. Diet diversity and the contributions of the main prey varied among breeding territories with different levels of forest cover. Black-and-chestnut Eagles nesting in more intact forests had a more diverse diet and fed primarily on mammalian carnivores. The South American Coati made the greatest biomass contribution to the diet of the eagle, which is a prey species that occupies mainly forested areas and forages predominantly in the canopy^52,53^. Whereas the biomass contribution of wild Galliformes such as Sickle-winged Guans and Andean Guans in the Black-and-chestnut Eagle diet is also higher in sites with low forest cover (Fig. 5). These species tolerate partly disturbed areas and deforestation, and Andean Guans are frequently observed near human populations^54,55^. The proportion of primates in the eagle diet also increased in sites with low forest cover. Forest degradation may intensify predation on canopy-dwelling primates by facilitating access to prey due to a lack of large trees where the presence of other mammals can be lower^56–58^.

Predators can be classified as generalists if they consume a wide range of prey, or specialists if they consume a narrow range^59^. Black-and-chestnut Eagles are preying on a very broad range of differently sized vertebrates (46 species) from 0.07 to 6.8 kg. This wide range of diet patterns indicates that, currently, the species has a generalist diet in the northern Andes. Furthermore, the standardized Levins food-niche breadth values of the eagle ranged from 0.19 to 0.88 (Supplementary Table 3), which suggests a wide variation in the selection for certain prey types on the part of the species, probably as a function of what is available in landscapes modified by humans such as the Andean rural landscapes of Ecuador and Colombia. Thus, the hunting behavior of the Black-and-chestnut Eagle suggests a flexible response to alternative prey, which probably makes the species more adaptable to changing environmental conditions. The long-term persistence of predators may depend on their dietary flexibility^12^. Species that can occupy a wide niche range are more likely to survive in human-modified habitats than highly specialized species^13,60^.

A better understanding of human impacts on the feeding habits of Black-and-chestnut Eagles requires quantifying the abundance of wild and domestic prey populations and evaluating the eagle’s prey preferences. Furthermore, studies on the Black-and-chestnut Eagle’s diet during non-reproductive periods should be conducted to examine whether the feeding on certain prey is only related to the need for parents to capture alternate prey such as guans and potentially also chickens to complement their diet and that of their chicks. This presents new challenges to gathering ecological knowledge for the conservation of Black-and-chestnut Eagles since studying the abundance of prey and diet of raptors in non-reproductive periods can be more complex due to the difficult logistics of sampling different types of prey and recording the feeding events that occur over much larger areas^61–63^.

Additionally, it is necessary to remember that diet studies of low-abundant large raptors encompassing data from different nests on a large spatial and temporal scale may have certain limitations when analyzing the data. For instance, when considering information from many nests, it is not always possible to collect the same amount of data during comparable periods or climatic seasons, which can restrict the type of analysis that can be performed and the extent of inferences. The use of a range of different techniques to study the diet of little-known large raptors during reproductive periods, such as collection of prey remains at nests, analysis of pellet contents, and use of nest cameras^32^, in combination with direct observations could contribute to increasing the sample size and to improving findings and conservation management measures.

To our knowledge, at least 40 Black-and-chestnut Eagle nests have been located in Ecuador and Colombia during the last decade. However, because of the complex topography, some of these nests were not logistically accessible to allow for direct observations or the use of nest cameras. Additionally, due to funding and technical constraints, our analysis was focused on 16 eagle nests, and the sampling period was relatively long (between 2017 and 2023). We acknowledge that our study had some limitations. However, our analysis provides useful information on the human conflicts over the Black-and-chestnut Eagle diet and the overall variation in the feeding habits of the species in relationship to forest cover in the northern Andes.

### Management implications

High predation rates on chickens in the northern Andes (Fig. 4, Supplementary Table 3) have contributed to the current human-Black-and-chestnut Eagle conflict^8,10,11^ and subsequent shooting and capture of the species in response to poultry predation^9^. Breeding territories in rural Andean landscapes in which eagles are persistently persecuted can create ecological traps (i.e., areas with easy and abundant prey but which are unsafe for predators)^18^ for Black-and-chestnut Eagles, which in turn may lead to the rapid decline of local populations in Ecuador and Colombia. Poaching due to human conflicts over wildlife, actual or perceived, is an important threat to large raptors worldwide^9,64–68^, such as Crowned Eagles^69^, Harpy Eagles (*Harpia harpyja*)^70^, and Andean Condors (*Vultur gryphus*)^65,66^, even resulting in species extinction^67^. Raptors provide important ecosystem services by controlling pests in crops and urban areas and cleaning the environment of organic material^71–73^. Local population extinction and decline of predators due to anthropogenic threats may result in trophic cascades, with severe consequences for human well-being^14,15,74–76^.

Our results reveal novel insights into the diet of this globally Endangered avian top predator and add evidence to support the effect of forest cover on raptor foraging strategies more broadly. Even though forest cover was not a factor that affected the consumption of poultry (i.e., domestic Galliformes), the Black-and-chestnut Eagle is an adaptable generalist able to switch from mammalian carnivores to guans (i.e., wild Galliformes) in human-dominated landscapes, and eagles nesting in sites with low forest cover had a less diverse diet than those in areas with more intact forests. Implementation of management actions to foster a more diverse diet for the species should include maintaining and increasing forest cover for the wild prey of the eagle using landscape management tools^77^ best suited to the particular social-ecological contexts of the eagle breeding territories in rural Andean landscapes. Furthermore, coatis and guans are illegally hunted by Andean rural people for food or their pelts, and, therefore, it is important to control the hunting pressure on these animals in the eagle’s breeding sites^7,8^.

Management of human–wildlife conflicts requires studies about the Black-and-chestnut Eagle's diet in areas where human persecution is suspected or documented. However, poultry predation is not the only predictor of the conflict with eagles in the northern Andes. Human conflicts over the Black-and-chestnut Eagle in Ecuador and Colombia are influenced by socio-demographic (i.e., gender, chicken ownership) and psychological factors (i.e., perceived detriments) but also by the disapproval of top-down local management, and people's perceptions of the species were largely negative^8,11^. Therefore, a combined social and ecological systems approach should be enacted to manage negative human-eagle interactions efficiently^78–80^. Even though each case of conflict is context-specific, multidimensional, and complex^81^, and requires a customized solution, the best way to scale up human–wildlife conflict mitigation is by using a community-focused conservation approach^18,82^. Necessary conservation measures should include the strengthening of technical capacities of rural communities, such as appropriate poultry management. This will help reduce their exposure to avian top predators by using enclosures and implementing bird-watching tourism projects in active eagle nests^8,9,83^.

### Supplementary Information

Supplementary Table 1.

Supplementary Table 2.

Supplementary Table 3.

Supplementary Table 4.

Supplementary Table 5.

## Data Availability

Data is provided within the manuscript or supplementary information files.
